# Medical Conditions of Nursing Home Admissions

**DOI:** 10.1186/1471-2318-10-46

**Published:** 2010-07-14

**Authors:** Gilberte Van Rensbergen, Tim Nawrot

**Affiliations:** 1 Department of Public Health, KULeuven, Leuven, Belgium; 2 Centre for Environmental Sciences, Hasselt University, Diepenbeek, Belgium

## Abstract

**Background:**

As long-term nursing home care is likely to increase with the aging of the population, identifying chronic medical conditions is of particular interest. Although need factors have a strong impact on nursing home (NH) admission, the diseases causing these functional disabilities are lacking or unclear in the residents' file. We investigated the medical reason (primary diagnosis) of a nursing home admission with respect to the underlying disease.

**Methods:**

This study is based on two independent, descriptive and comparative studies in Belgium and was conducted at two time points (1993 and 2005) to explore the evolution over twelve years. Data from the subjects were extracted from the resident's file; additional information was requested from the general practitioner, nursing home physician or the head nurse in a face-to-face interview. In 1993 we examined 1332 residents from 19 institutions, and in 2005 691 residents from 7 institutions. The diseases at the time of admission were mapped by means of the International Classification of Diseases - 9th edition (ICD-9). Longitudinal changes were assessed and compared by a chi-square test.

**Results:**

The main chronic medical conditions associated with NH admission were dementia and stroke. Mental disorders represent 48% of all admissions, somatic disorders 43% and social/emotional problems 8%. Of the somatic disorders most frequently are mentioned diseases of the circulatory system (35%) [2/3 sequels of stroke and 1/5 heart failure], followed by diseases of the nervous system (15%) [mainly Parkinson's disease] and the musculoskeletal system (14%) [mainly osteoarthritis]. The most striking evolution from 1993 to 2005 consisted in complicated diabetes mellitus (from 4.3 to 11.4%; p < 0.0001) especially with amputations and blindness. Symptoms (functional limitations without specific disease) like dizziness, impaired vision and frailty are of relevance as an indicator of admission.

**Conclusion:**

Diseases like stroke, diabetes and mobility problems are only important for institutionalisation if they cause functional disability. Diabetes related complications as cause of admission increased almost three-fold between 1993 and 2005.

## Background

The reason for admission to long-term nursing home (NH) care is often a combination of factors. Andersen & Aday presume that use of a nursing home is a function of three classes of variables: personal attributes that predispose individuals to seek care; enabling factors that influence access to care and need factors as reflected by health status, disease and functional disability [1]. Jette et al. examined the predictive power of 11 predisposing, 7 enabling and 18 need factors. The findings illustrated that restricted outside mobility and basic Activities of Daily Living (ADL) were the strongest individual predictors [2].

In the 1990s/2000s there have been a large number of studies that have examined predictors of nursing home placement. The most commonly identified personal risk factors include advanced age, levels of P-ADL and I-ADL (Personal and Instrumental Activities of Daily Living), mental impairment, living alone and the presence of specific medical conditions. Studies have also shown the influence of gender (women), race, existential problems (feeling lonesome), impaired vision and balance (dizziness) [3-8].

In Belgium, the admission criteria for NH residents focus on the degree of functional limitations that people experience but medical admission grounds are often vague or missing in the resident's file. Mostly one states multimorbidity data next to medical history.

Some countries even pay special attention to the registration of these medical conditions. The United States National Nursing Home Survey provides data about functional dependencies in ADL's and IADL's, but also primary and other diagnoses at admission and noticed that the leading admission diagnoses for elderly nursing home residents were diseases of the circulatory system, followed by mental disorders [9]. Banaszak et al. demonstrated significant effects of chronic medical conditions and dementia on the risk of institutionalization [10]. According to Chan, the most common medical problems of the applicants were neurological, heart diseases, orthopaedic conditions and psychiatric problems" [11]. McNabey et al. quantified four chronic medical conditions: congestive heart failure, chronic obstructive pulmonary disease, diabetes mellitus and Parkinson's disease [12].

Avoiding disease and disability can avoid NH admission. To know the medical risk profiles is very useful for developing interventions to prevent the need for institutionalisation [2].

For instance well treated diabetes, stroke and mobility problems may in some cases remove the functional limitations. Primary care providers can modify the pathology and mitigate the expression of disease through prevention and treatment [13].

The major emphasis of this exploratory study is to describe the real underlying diseases causing the disabilities (= the 'reviewed' primary diagnoses at admission) and to explore the evolution over a period of twelve years.

Medical issues surrounding nursing home care are becoming increasingly important for public health policymakers from the point of view of health education, and to primary care providers for prevention, detection and treatment of people who are at risk of chronic medical conditions.

## Methods

The aim of this exploratory survey was to quantify and characterize the chronic conditions of NH applicants who are lower functioning. In Belgium, NH beds, legally created in 1982 and destined for elderly in need of care, were primarily set up in the lap of existing rest homes (RH) who accommodate valid elderly, at an average of 12 NH beds out of 100 RH beds.

Since 2000 the care setting in Belgium was comparable with the international criteria for nursing homes (± 80% of the residents are in severe need of care)[12].

Residents which are defined as 'in need of care' are to a certain extent dependent on ADL. Dependence in ADL (washing, dressing, transferring, toileting, incontinence and feeding) was used to classify residents' physical functional status. The psychical items include disorientation in time and place. The resident classification scale distinguishes four main categories of ability. Residents of category O don't need assistance with any ADL's; category A need assistance in two ADL's (washing and dressing); category B require assistance in three and category C in more than three ADL's. The subcategories Bd and Cd are people in need of care, who are also disorientated.

This study is based on two independent, descriptive and comparative surveys (field research) conducted in 1993 and 2005. Data from the subjects were obtained from two convenience samples. The first sample survey was an unpublished part of a pilot project under the authority of the Belgian Ministry of Health. In 2005 we performed an update.

The questionnaire, with structured and open ended questions, was derived from the traditional hospital questionnaire that collects medical data. Based on the residents' medical records, socio-demographic variables were filled in by the head nurse. The primary diagnosis was written in full (open question) by the general practitioner at the moment of admission. The gathered data were mapped by means of the International Classification of Diseases - 9th edition (ICD-9) [14] by trained staff of the University Hospitals of Leuven. In 1993 it involves all the 12 000 diagnosis codes. We used the same classification in 2005, yet with a simplified encoding system. In 1993 seven secondary diagnoses could be given, in 2005 only one. The study was performed in accordance with the Helsinki Declaration and was approved by the ethical board of nursing home organizations.

In 1993 all current and deceased NH residents during the first trimester, were included. It involves 1,332 residents (338 men and 994 women) from 19 institutions.

The cross sectional survey of November 2005, (n = 691; 151 men, 540 women) involves seven representative institutions, namely six comparable NHs and one smaller institution (Cocoon), exclusively specialised in the care of people having dementia. Residents with a short stay were excluded.

The primary diagnosis (reason of admission) constitutes together with a possible 'secondary diagnosis', (the major pre-existing disorder), the medical condition of NH residents at the time of admission (point prevalence). We gathered information in both medical files and open interviews with the nursing home physician or the residents' general practitioner and/or the head nurse to determine the *true medical reason *why the elderly applied for NH admission.

Longitudinal changes in reason for admission were assessed and compared by a χ^2^-test.

The residents were divided in two groups; those with a somatic reason for admission and those with a mental illness.

## Results

### 1. General characteristics of the NH population

In 1993 the mean age at admission was 83.2 years (SD 8.0) and 75% were women. The corresponding numbers in 2005 were 84.4 years (SD 8.3) and 78%.

In 2005 more (proportional) details were available (table 1). Somatic disorders represented *43% *of all admissions, mental disorders *48% *and social/emotional problems (SEM) *8%.*

**Table 1:** Characteristics of the population in the various Nursing homes (2005)

Institutions	Bed size	Katz category (%)						Men %	Women %	Cause of admission %			Average Age (SD)
		**O**	**A**	**B**	**C**	**Bd**	**Cd**			**Somat**	**Mental**	**Soc/Emo**	

Cantershof	100	7	14	23	15	0	41	26	74	33	59	8	84.6 (7.3)

Cleo	90	6	20	20	10	0	44	12	88	37	53	6	87.5 (7.5)

Cocoon	51	2	10	18	4	0	67	31	69	2	98	0	83.5 (9.1)

Compostela	98	12	9	3	15	22	38	31	69	58	39	3	84.8 (7.3)

Czagani	104	13	11	16	19	0	41	14	87	40	48	12	83.9 (8.1)

Den Olm	159	5	13	19	20	0	44	20	81	51	39	10	83.8 (9.2)

Releghem	89	11	10	12	23	7	37	26	74	30	60	10	82.6 (8.1)

**Total**	**691**	**8**	**12**	**16**	**16**	**4**	**43**	**22**	**78**	**40**	**52**	**8**	**84.4 (8.3)**

Legend: category O: residents who don't need assistance with any ADL's.

category A: residents who need assistance in two ADL's (washing and dressing).

category B: residents who need assistance in three ADL's.

category C: residents who need assistance in more than three ADL's.

category Bd or Cd: residents category B or C who are also disorientated.

The number of residents with a mental disorder ranged from 39 to 60% per institution (Cocoon 98%). Women were the large majority with an average of 78%, from 69 to 88% per institution. In all institutions, approximately 20% was 'higher functioning', this means category O/A and 43% of the residents were located in the most severe category Cd.

### 2. Description and evolution of the somatic primary diagnoses

Classifying the physical disorders according to the affected system, in accordance with the ICD-9, we get table 2 in decreasing order (according to 1993).

**Table 2:** Classification of the somatic primary diagnoses per disease system: 1993 (n = 688); 2005 (n = 278)

Disease systems	ICD-9	% 1993	% 2005	Most frequent disorder per section (%)
10. Circulatory system	390-459	36.3	35.4	Stroke (64, **66**), heart diseases (12, **21**)
11. Nervous syst/sense org.	320-389	21.3	15.5	Parkinson (35, **45**), hemiphlegia (30), blindness (**21**)
12. musculoskeletal system	710-739	13.0	14.0	Osteoarthritis (46, **26**), fractures
13. Neoplasm's	140-239	5.5	3.0	intestinal(24)-prostate cancer(26)
14. Endocrine disorders	240-279	5.5	11.4	Diabetes (87, **100**)
15. Accidents	800-999	4.8	5.9	(femur)Fractures (64, **56**), head injuries, CO-poison.
16. Respiratory system	460-519	3.8	1.9	COPD
17. Genitourinary system	580-629	2.9	2.6	Renal insufficiency NEC (80, **100**) en nephropathy
21. Post surgical state	V-code	1.9	3.3	Stoma (APN), hipprosthesis(54, **33**), amputation(**56)**
20. Symptoms	780-799	1.9	4.1	Abnormality of gait
18. Digestive system	520-579	1.6	0.4	Stomach-liver disorder
19. Diseases of blood	280-289	0.6	1.1	Anaemia
22. Diseases of skin	680-709	0.6	-	Chronic ulcer
23. Infectious diseases	001-139	0.3	1.5	Late effects of poliomyelitis

**Total**		100%	100%	

The numbers in standard between brackets indicate the percentage in 1993, while those in bold refer to the percentage of 2005. For instance, in 1993, strokes represented 64% of the disorders of the circulatory system and in 2005 66%. For small absolute numbers, no percentage was calculated.

The table showed a comparable relative frequency of the different disease systems between 1993 and 2005 (except diabetes).

For 1993 and 2005, disorders of the circulatory system were the most widely indicated primary diagnosis (36.3 vs 35.4% respectively; p = 0.78), of which two thirds were strokes, followed by disorders of the nervous system (21.3% vs 15.5%; p = 0.04), of which one third was Parkinson, and the musculoskeletal system (13% vs 14%; p = 0.03). Two thirds of the strokes was attended with hemiphlegia, the others had slighter sequels. More men were affected (36 versus 17) but women were often affected when they are younger (mean age 79.2; SD 12.1 versus 80.9 year; SD 6.9 for men).

The chronic heart diseases increased from 12 to 21% (p = 0.07) and the fraction of osteoarthritis declined from 46 to 26% (p = 0.14). The most striking evolution is by diabetes type 2, which was indicated as a primary diagnosis in 11.4% of all admissions in 2005 versus 5.5% in 1993 (p = 0.0001). Moreover, the diagnosis often went hand in hand with serious related problems like amputations, polyneuropathy, kidney failure and blindness.

The decrease of the percentage of disorders of the nervous system was mainly due to the fact that hemiphlegia was no longer registered separately in the nervous system.

Ex-poliomyelitis patients remain represented (two in 1993 and four in 2005) and 'symptoms' were still indicated as reason for an admission.

In 2005, we remarked the disappearance of skin diseases as well as a regression of cancer (p = 0.08) and COPD (p = 0.11). In 2005 two cases of eye cancer were still admitted because of blindness.

Besides MS and ALS, we also noticed an increasing number of (sub) coma patients and posttraumatic victims in some facilities.

### 3. Description and evolution of the mental primary diagnoses

In the ICD-9, the mental disorders were categorised in psychosis, neurosis and mental retardation (table 3). Dementia remains the most important one (95% of the psychosis group). An increase of the psychiatric disorders was remarkable (p = 0.01). Mainly paranoia and schizophrenia were mentioned. In 2005, 17 residents (out of 360) were admitted with a psychiatric primary diagnosis, compared to 11 (out of 636) in 1993.

**Table 3:** Classification of mental primary diagnoses per disease group: 1993 (n = 636); 2005 (n = 360)

Disease group	ICD-9	% 1993	% 2005	Most frequent disorder per group (%)
Psychosis	290 - 299	97.3	94.0	Dementia (98, **95**), Psychiatric disorder (2, **5**)
Neurosis	300 - 316	1.9	3.9	Depression(42, **64**), alcohol abuse, behavioural dist.
Mental retardation	317 - 319	0.8	1.7	Mental retardation

**Total**		100%	100%	

The numbers in standard between the brackets indicate the percentage for 1993; those in bold refer to 2005.

In the neurosis group, depressions have risen from 42 to 64% (p = 0.03), or from 5 cases in 1993 to 9 in 2005. Five residents in 1993 and six in 2005 were admitted because of mental retardation.

In 1993, little distinction was made between the different types of dementia. In 2005, we noticed a more specific classification of the mental disorders (table 4).

**Table 4:** Classification of the mental primary diagnoses per specific disease and per institution (2005)

Code	30	31	32	33	34	35	36	39	40	41	42	43	45	
**Institutions**	Dem	DAT	SDAT	MID	S dem	Kor	DLB	Psy	Neu	Depr	Abus	Behav	Retar	**Tot.n**

Cantershof	36%	17	0	3	19	7	3	7	0	8	0	0	0	59

Cleo	35	29	13	2	2	6	0	4	0	4	2	2	0	48

Cocoon	10	60	6	2	18	2	0	2	0	0	0	0	0	50

Compostela	0	3	0	3	79	6	0	8	0	0	0	0	3	38

Czagani	2	80	0	10	0	2	0	2	2	0	0	0	2	50

Den Olm	15	5	53	6	6	3	2	2	0	3	0	0	5	62

Releghem	64	6	0	4	0	11	0	9	0	0	0	4	2	53

**Total, %**	**24**	**28**	**12**	**4**	**15**	**5**	**1**	**5**	**0.3**	**3**	**0.3**	**1**	**2**	

**Total, n**	**87**	**101**	**42**	**16**	**55**	**19**	**3**	**17**	**1**	**9**	**1**	**3**	**6**	**360**

**Code 30 to 39: psychosis **(code 290 - 299 in the ICD-9)

30 = Dementia NEC; 31 = Dementia of the Alzheimer Type (DAT); 32 = Senile Dementia of the Alzheimer Type (SDAT); 33 = Multi-infarct dementia (MID); 34 = senile dementia; 35 = specific dementia (Korsakoff, Huntington); 36 = Dementia with Lewy Body (DLB); 39 = Psychiatric disorder (mainly paranoia, schizophrenia)

**Code 40 o 43: neurosis **(code 300 - 316 in the ICD-9)

40 = Neurosis NEC; 41 = Depression; 42 = abuse (alcohol); 43 = behavioural disturbance.

**Code 45 = mental retardation **(code 317-319 in the ICD-9)

Looking only at dementia and its subtypes (n = 323; code 30-36), the following percentages emerged: Dementia NEC 27%; (Senile) Dementia of the Alzheimer Type (code 31 + 32) 44%; MID or Vascular dementia 5%; Senile dementia 17%; Korsakoff/Huntington 6%; DLB 1%.

The concepts of 'dementia NEC' and 'senile dementia' continued to be mixed up. The distinction between 'dementia type Alzheimer (DTA) and senile dementia type Alzheimer (SDTA) remained vague. We highlight the following statistics. The institution Compostela had one resident with DTA, versus 30 with SDTA. Exactly the opposite could be noticed at Czagani where mainly DTA was encoded. Releghem usually registered dementia NEC.

Psychiatric disorders were present in all the institutions. In case of neurosis, only depression was important. Four institutions accepted feeble-minded (mostly less aged) elderly people.

### 4. Description of the secondary diagnoses

In 1993, seven secondary diagnoses could be registered. In no hierarchical order, anamnesis data as well as geriatric and common complaints, like insomnia and constipation, were mentioned. Symptoms of the disease itself were additionally recorded, as for instance 'confusion' in case of dementia. Hip fractures, hip replacements and walking difficulties provided three codes. This resulted in a complex and unclear arrangement.

In 2005, only one secondary diagnosis could be given, which happened for six out of ten residents. A secondary diagnosis could only be given, if it was truly important as a pre-existing disease at the time of admission.

Especially people having dementia had only dementia as a diagnosis, both in 1993 and in 2005. A possible secondary diagnosis mentioned striking behavioural disturbances. For people in need of somatic care, usually symptoms and disorders of the circulatory system, mainly high blood pressure, were registered as a secondary diagnosis in 1993.

In 2005, 418 secondary diagnoses were quoted: 70% of physical nature and 30% of mental nature.

In the field of somatic secondary diagnoses, symptoms scored the highest (22%), by which we mean 'frequently falling' and 'abnormalities of gait', followed by diabetes (20%). Next, we came across disorders of the nervous system and sense organs (14%), mostly blindness, followed by post surgical status (13%), in particular amputations, stomas and hip prostheses, and finally heart failure (10%) and osteoarthritis (10%).

For the mental secondary diagnoses, mainly beginning or alleged dementia was indicated (36%), as well as behavioural disturbances (36%), primarily 'disturbing' shouting behaviour. Depressions constituted a secondary diagnosis in 12% of the cases and in 8% of the cases this was an additional psychiatric problem.

## Discussion and conclusion

### 1. Principal findings and their interpretations

#### 1.1. Dementia and stroke are most associated with NH placement

Fourthy-three percent of all admitted residents have dementia (95% of the psychosis group) and 36% of the residents with somatic problems have disorders of the circulatory system, mostly a stroke. The relatively high frequency of these disorders in NH facilities is consistent with other research [15-17].

In Belgium and in Europe, the prevalence of a certain form of dementia increases and grows exponentially with age but precise figures on the number of formally identified cases are missing [18,19]. In 2005 Ferri et al. estimated the dementia prevalence for Europe at 25% for elderly ≥85 years. [20].

In our study dementia seems to affect particularly the oldest among the old, as well as women. People with dementia had an average age of 85 years (slightly above the average of the total group) and were mostly female (81%). The observation that dementia 'age-, and gender-related' is, is very similar with other research [15,20-23].

The number of institutionalized dementia sufferers might be a good representation of the number of patients in the community who suffer from a moderate to severe form of dementia.

A study, regarding a mortality cohort in Belgium in the year 2000 [6], mentioned that 8 out of 10 deceased patients with dementia have resided in a NH before they die.

#### 1.2. NH residents have their own health profile with fixed components

According to our study, residents seem to have their own health profile, with fixed components, that has shown minimal changes over the last twelve years. In all participating facilities, we observed roughly the same range of some 100 disorders. Dementia, disorders of the circulatory system (e.g. stroke, heart failure), the nervous system (e.g. hemiphlegia, Parkinson) and the musculoskeletal system (e.g. osteoarthritis, femur fractures) continue to form the core of the diseases common in NHs. This diagnosis ranking corresponds to what was found in literature [11,24].

#### 1.3. Increasing of Diabetes Mellitus (1993 vs 2005)

The prevalence of diabetes is rising all over the world and this tendency can be seen in the NHs. In our data the most striking shift in prevalence across the 12 year period was the increased number of diabetics, especially because of the related complications.

A Belgian national report shows the strong amount of diabetes related diseases. The prevalence of most complications doubled at least, and the prevalence of amputations and blindness is even 4 times higher in 2007 than in 2002 [25].

Other studies similarly mention diabetes as an important disease associated with new admission to a nursing home [26,27].

Diabetes strongly reduces the quality of life in advanced age. The complications for other organs, mainly circulatory problems, neurological problems, retinopathy and nephropathy, do not only affect the mind (leg amputations, blindness, dialysis), but they also drastically diminish the ability to cope. All actions have to be focussed on health education, prevention, detection and treatment. *Diseases like diabetes (and stroke) only are important for institutionalisation if they cause functional disability*. If these diseases are treated well, their consequences will be less severe. Banaszak et al. showed that the effects of stroke and diabetes on institutionalization disappear after controlling for functional disability [10].

#### 1.4. The necessity of an appropriate and concise encoding system

The entire disease classification for NH residents included some hundred typical disorders. Using the 12 000 diagnostic codes of the ICD-9 is difficult and unpractical in a NH context. Abstracted from this extensive clinical instrument, an 'adapted and shortened' classification of the diseases was used in 2005 and was as reliable and effective as his longer counterpart in 1993. Short versions of instruments are often as good as their longer equivalents [23,28,29]. Such a condensed version allows an optimal accuracy and comparability.

### 2. Strengths and limitations of the study

#### 2.1. Definition and selection of the real primary diagnosis

A standard interpretation of the primary diagnosis is very important. We defined this diagnosis as the motive for admission. The medically most severe condition is not necessarily the primary diagnosis. One should always ask oneself: 'Is this the real reason for admission? We sketch a few cases.

- An elderly person, who suffers from a severe form of diabetes, does not need to be admitted, even if he lives on his own. The situation changes if this person starts to be confused and if he is no longer in control of his eating habits or taking his medication. The direct cause of admission in this case is 'starting dementia' and diabetes constitutes the secondary diagnosis.

- A 91 year old woman with a leg amputation has been living at home for a while. Confusion and an alleged beginning dementia cause the family to send her to a nursing home.

- An 84 year old man with an official diagnosis of 'prostate cancer' is admitted because of disturbances of equilibrium after the death of his wife. He was afraid to fall and not to be able to raise the alarm.

Since the survey was performed in close consultation (face to face interview) with the general practitioners or nursing home physicians and the head nurses, it was easier to select the clinical primary diagnosis, as the underlying disease causing the disabilities.

When discussing the primary diagnoses, the degree of reliability and severity of the diseases intrinsically was taken into account.

#### 2.2. Special attention to symptoms and social/emotional issues

Mostly symptoms were quoted as secondary diagnoses in our sample. Disease related symptoms were usually not accepted as a primary diagnosis; the underlying disease had to be indicated. For instance, 'difficulties of walking' can occur in case of dementia, hip prosthesis, osteoarthritis, rheumatism, Parkinson's disease, poliomyelitis or bone TBC. Dementia, brain contusion, traumatic events in the family or psychiatric problems can cause severe behavioural difficulties.

Symptoms without an identified cause, yet with a large impact on disability, may well be a primary diagnosis, for instance dizziness, lack of standing function without clinical labelling.

Social/emotional problems were not mentioned in 1993, and also in 2005, this admission indication was only taken into account after discussion. The main reasons of a SEM admission were 'advanced age' without a specific clinical disorder; being admitted together with a husband or wife; extreme social neglect; loneliness or weariness of life after the loss of a dear one.

#### 2.3. Specifying dementia remains difficult

In 1993 we were not able to classify adequately the mental disorders. People having dementia were primarily (428 cases or 71%) encoded as ' psychosis NEC' (Not Elsewhere Classifiable or unspecified) and 'senile dementia' (16%). On the other hand, one used an abundance of terminology to indicate dementia. Patients with 'unspecified cerebral degeneration' (code 331) or 'encephalopathy' (437.2), which come under the nervous system and the circulatory system respectively, according to the ICD-9, were residents with dementia in the NH practice.

In the ICD-9 alphabetic index, Alzheimer dementia is classified under 'presenile dementia' (290.1), but is also encoded as 331.0 'other cerebral degeneration'.

No real distinction was made between 'depression NEC' (311), which belongs to the neuroses, and 'major depressive disorder' (296), which belongs to the psychoses.

The number of people identified as demented according to the disability criteria (scores above the screening cut-off point for dementia) can differ greatly from the number of people according to medical standards (clinically diagnosed dementia)[23]. Cognitive impairment can have other causes than dementia (i.e. delirium, Parkinson).

Even in the year 2000 some elderly people, diagnosed as having dementia, ended up in an institution, while afterwards their problem turned out to be a reversible temporary confusion such as medication intoxication [30].

As specific dementia-diagnoses are missing in the 1993 population, comparing the differences with 2005 was difficult. The literature confirms that fifteen years ago 'confusion' was easily labelled as 'dementia' [31]. Since that time, numerous publications about the sensitivity of testing and guides for psychopathology have come out [32].

The clinical practice of diagnostic refinement has not established itself in the NHs. Until now, most of the residents' files still mention (beginning or alleged) 'dementia NEC'. *Differential diagnosis *remains problematic and may consequently cause some misclassifications.

Epidemiological studies of dementia subtypes have revealed widely varying distribution rates [33]. Recently, in May 2009, the European Collaboration on Dementia (EuroCoDe) project [22] aimed at producing new consensual guidelines, hoping to achieve a better understanding of the breakdown between various forms of dementia.

Alzheimer's disease and vascular dementia are the most frequently identified subtypes respectively 69% and 25% [Erkinjuntti et al. in Stevens, 23]. Dementia with Lewy bodies (DLB) is a relatively recently identified entity and changing neuropathological and clinical diagnostic criteria have resulted in great variation in the frequency, ranging from 1.7% to 30.5% (respectively Herrera and Stevens in Zaccai et al.) [34]. Regarding the frontal lobe dementias (FLD), there is a dearth of epidemiological data. In Stevens et al. [23] Oliva estimated the distribution of this subtype in 2000 at 10-20% but in 2001 Yamada identified no cases.

Our data quality was influenced by the fact that not only mental health professionals like psychiatrists or neurologists were making the diagnosis. It is highly likely that many of our figures on the distribution of dementia subtypes are forced into 'unspecified dementia'.

#### 2.4. The lack of a (concise) standardised instrument fulfilling our purpose

On the one hand the coding difficulties that arose in 1993 are situated in the field of coding possibilities. The ICD-9 did not specify sufficiently common disorders of NH residents. For instance, 'Advanced age' is classified under 797: 'senility'. Medical diagnoses are mostly described by means of the first three coding numbers, a fourth and fifth number is optional. Yet, Alzheimer or vascular dementia can only be indicated with four digits. Combined codes, like the ones used in hospitals to indicate (one) primary diagnosis, are statistically difficult to process.

On the other hand, the inaccurate diagnosis descriptions, caused some diseases to occur in various disease classification systems or in the category 'NEC'. For instance: 'Mobility problems' could be encoded as '728.3: immobility syndrome', or '781.2: abnormalities of gait' or '719.7: walking difficulties'. In that case, the problem belongs to muscular disorders (728), to symptoms (781) or to joint disorders (719).

Schnelle ascertained in two nursing homes, with an unusually high and low depression frequency respectively, that the prevalence of depression reflects the measuring process rather than the results [35].

In 2005 we categorized the diseases by means of an adapted and shortened version of the ICD-9 but it was not a standardised instrument. Available standardised instruments i.e. The Resident Assessment Instrument (RAI) measure particularly the need of care but the possibilities for medical diagnostic are limited. Although the increased coding possibilities of the ICD-10 (75.000 codes), there is certainly a need to create a more appropriate and statistical optimal manageable classification system (abstracted from e.g. ICD-10). Such an assessment tool can be used to achieve a 'medical databank' for nursing homes on a rapid, reliable and efficient way.

Nihtilä also used own Finnish codes next to the ICD-9 [36].

#### 2.5. Statistics on medical diagnoses only, can have merits

The true reason for admission to long-term care is often a combination of medical diseases and their consequences on function, together with other patient related and social factors. To subtract and study the medical diagnoses alone is normally difficult.

Although such an approach can have an influence on the reasons of admission, we did not take it into account because we could expect a similar effect of the other (personal/social) variables for all the diseases.

Dementia, Parkinson's disease, stroke, depressive symptoms, hip fractures and diabetes are strongly associated with increased risk of institutionalization, *independent *of socio-demographic confounders and service use[36].

### 3. Strengths and weaknesses in relation to other studies

While existing research has identified significant predictors of NH admission, the present analysis attempted to provide more empirical findings (case analysis of each resident without refusals) to identify in detail the underlying disease of the disability. The real medical reason of admission (reviewed primary diagnosis in a face to face interview) was coded according to the ICD-9. The use of this coding system is unique for NHs.

Some studies carried out secondary analyses and/or focused entirely on residents with dementia [37] or with a restricted number of medical problems [12] or with dichotomous indicators [36].

The weakness of our study concerns the method used and the poor results obtained in our 1993 sample, specially due to disagreement about the terminology used to describe the diseases. As we have no specific data on dementia in 1993 we only could compare the somatic disorders. Although the method and the design are not standard, it was the best to achieve this research. Further, we did not explain the causes of changed prevalence rates like others [38].

## Conclusion

The future demand for institutional care depends not only on the aging of the population but also on the prevalence of chronic medical conditions, especially debilitating illnesses. All actions which improve the health status and functional capacity of elderly people contribute to decrease the need for institutional care.

Our results highlight the importance of an effective prevention, detection and treatment of people who are at risk of, or suffer from diseases like stroke, diabetes and mobility problems. These risk profiles constitute a challenge for policy makers, primary care providers and hospitals to delay or prevent institutionalization.

Need factors have the greatest impact on NH admission but the underlying diseases causing these disabilities are not well studied. This requires accuracy of diagnosis and standardisation of diagnostic criteria together with an appropriate and user-friendly classification system to achieve both somatic and mental disorders. The ability to distinguish particular subtypes of dementia is a key issue.

## Competing interests

The authors declare that they have no competing interests.

## Authors' contributions

GVR designed the study, carried out the interviews and wrote the first draft of the paper. TN performed the statistical analysis. All authors read and approved the final manuscript.

## Pre-publication history

The pre-publication history for this paper can be accessed here:

http://www.biomedcentral.com/1471-2318/10/46/prepub
